# Pediatrics Specialty Choice Among Matriculants of MD-Granting US Medical Schools

**DOI:** 10.1001/jamanetworkopen.2026.0603

**Published:** 2026-03-04

**Authors:** Amy Pineda, Robert J. Vinci, Adam Turner, Dorothy Andriole, Douglas Grbic, Mytien Nguyen, Patricia Poitevien

**Affiliations:** 1 Medical Education Research, Association of American Medical Colleges, Washington, DC; 2 Chobanian and Avedisian School of Medicine, Boston University, Boston, Massachusetts; 3 American Board of Pediatrics, Chapel Hill, North Carolina; 4 Yale University School of Medicine, New Haven, Connecticut; 5 Warren Alpert Medical School, Brown University, Providence, Rhode Island

## Abstract

**Importance:**

The numbers of US MD-granting school graduates choosing pediatrics have decreased over the past decade. Identifying variables associated with pediatrics specialty choice at graduation may inform efforts to strengthen the emerging pediatrics workforce.

**Objective:**

To describe the prevalence of, and identify variables associated with, pediatrics specialty choice at graduation among 7 US MD-granting school matriculant cohorts.

**Design, Setting, and Participants:**

This cohort study used deidentified, individual-level Association of American Medical Colleges (AAMC) data from 2013-2014 through 2019-2020 matriculant cohorts, followed up through 2022-2023, who graduated and responded to the AAMC Graduation Questionnaire (GQ) though 2022-2023. Data were analyzed from January 2025 to August 2025.

**Exposures:**

Demographic characteristics, medical school experiences, and career plans.

**Main Outcomes and Measures:**

The main outcome, measured with GQ data, was specialty choice at graduation, dichotomized as pediatrics vs all other specialties. The χ^2^ test and analysis of variance were used to assess bivariate associations. Multilevel logistic regression was used to assess associations with the outcome.

**Results:**

Of 101 579 GQ respondents in the study (75% of all 134 759 study-eligible graduates), 52 810 (52%) were women, 55 027 (54%) self-identified as White, and 10 031 (10%) chose pediatrics at graduation. Odds of pediatrics specialty choice at graduation were higher for women (vs men; adjusted odds ratio [AOR], 2.34 [95% CI, 2.23-2.46]), those with pediatrics (vs all other specialties) intention at matriculation (AOR, 9.21 [95% CI, 8.68-9.76]), those who rated the quality of their pediatrics clerkship more highly (per unit increase in quality; AOR, 2.22 [95% CI, 2.13-2.32]), and those whose career plans at graduation included (vs did not include) patient care (AOR, 2.19 [95% CI, 1.77-2.71]); odds were lower for 2019-2020 (vs 2013-2014) matriculants (AOR, 0.72 [95% CI, 0.66-0.79]), and for those whose career plans at graduation included (vs did not include) research (AOR, 0.75 [95% CI, 0.72-0.79]). In separate models for men and for women, associations were of similar direction and magnitude.

**Conclusions and Relevance:**

In this cohort study, pediatrics specialty choice at graduation decreased among more recent matriculant cohorts. We identified variables independently associated with pediatrics specialty choice at graduation, which can inform efforts before and during medical school to promote or sustain pediatrics specialty choice among US MD-granting school matriculants.

## Key Points

**Question:**

What is the prevalence of, and variables associated with, pediatrics specialty choice at graduation among US MD-granting school matriculants?

**Findings:**

In this cohort study of 7 US MD-granting school matriculant cohorts, 10% of matriculants chose pediatrics at graduation. Pediatrics specialty choice at graduation odds were higher for those who intended pediatrics at matriculation, rated their pediatrics clerkship quality more highly, and planned careers including patient care; odds were lower for more recent matriculants and those who planned careers including research, and, in models among men and among women, associations were similar.

**Meaning:**

These findings highlight opportunities before and during medical school to promote or sustain pediatrics specialty choice.

## Teaser

This cohort study describes the prevalence of, and variables associated with, pediatrics specialty choice at graduation among matriculants of MD-granting medical schools in the US.

## Introduction

The US pediatric population has become increasingly racially and ethnically diverse while maintaining a relatively steady national population of more than 73 million, for ages 17 years or younger.^1,2^ Simultaneously, children’s health outcomes have been declining.^3^ Attending to the health needs of the nation’s children provides the foundation for achieving a healthy and vital adult population^4^ and requires a well-trained pediatric workforce. The most recent National Resident Matching Program (NRMP) Main Residency Match resulted in the largest number of individuals ever matched into pediatrics,^5^ representing a 23% increase over 15 years.^6^ However, there have been substantial changes in the composition of NRMP participants matching to pediatrics positions,^7^ with decreasing numbers of senior students of US MD-granting schools (US MD seniors) matching in pediatrics. According to NRMP data, 1464 US MD seniors matched to categorical pediatrics positions in 2024,^5^ representing the lowest number of matched US MD seniors in pediatrics since 1994^5,6,8,9,10,11,12^ and a 22% decrease over the decade (2015: 1889 US MD seniors matched to categorical pediatrics positions).^6^ In contrast, from 2015 to 2024, the number of matched US MD seniors in family medicine and in categorical internal medicine each increased by 8%.^5,6^ Additionally, while the number of categorical pediatrics positions filled has increased, the overall fill rate decreased from 99.5% (2654 of 2668) in 2015 to 91.8% (2827 of 3078) in 2024.^5,6^ In response to these decreases, work by the Association of Medical School Pediatric Department Chairs Pediatrics Workforce Initiative,^13^ among others,^14,15,16,17^ has focused on aspects of undergraduate medical education (UME) and graduate medical education (GME). To date, however, there have been few investigations, including relatively small cohorts, into variables associated with students’ pediatrics specialty choice at medical school graduation.^14,15^ Education debt and its association with pursuing pediatrics careers has been a focus of 2 studies,^16,17^ including an examination among pediatric GME trainees.^16^

Coincident with the period of decreasing proportions of US MD students choosing pediatrics, the number of US MD-granting schools increased,^18^ as did total graduate numbers.^19^ In addition, the proportion of women, and those of racial and ethnic populations underrepresented in the medical profession (URiM) relative to their numbers in the general population, among US MD-granting school matriculants, steadily increased over the past decade.^20,21^ In this context, we sought to describe the prevalence of, and identify variables associated with, pediatrics specialty choice at graduation among 7 national cohorts of US MD matriculants who entered and graduated from medical school during this period of decreasing pediatrics specialty choice.

## Methods

This cohort study was deemed exempt from institutional review board review by the Association of American Medical Colleges (AAMC) Human Subjects Office because it did not involve human participants, as defined in 45 CFR 46, given the use of deidentified data. This study followed the Strengthening the Reporting of Observational Studies in Epidemiology (STROBE) reporting guideline for cohort studies.

We created a deidentified, individual-level dataset of our study-eligible population: all 2013-2014 through 2019-2020 US MD-granting school matriculants who graduated and were invited to complete the AAMC Graduation Questionnaire (GQ) through 2022-2023. The GQ, a national questionnaire annually administered to graduating students for voluntary participation, includes items on medical school experiences and career plans (among others).^22^ Informed by a literature review on US medical students’ specialty choice determinants,^14,23,24,25^ we examined the following variables in association with the study outcome.

Using AAMC American Medical College Application Service data,^26^ we included gender (men, women [other identities, representing less than 1% of the study-eligible population, excluded]), matriculation year, and self-reported race and ethnicity, which we grouped into 4 mutually exclusive categories: Asian (alone); URiM (each alone or in combination with another race or ethnicity: American Indian or Alaska Native; Black or African American; Hispanic, Latino, or of Spanish origin; and Native Hawaiian or Pacific Islander); White (alone); and all other or unknown (combinations of Asian, White, or other [selection of “other” response]; and missing).

Using AAMC Matriculating Student Questionnaire (MSQ) data,^27^ we created a 3-category variable for specialty intention at matriculation: pediatrics; all other specialties (those who selected any other specialty choice); and missing. Using AAMC GQ data, we created our dichotomous outcome for specialty choice at graduation: pediatrics (those who selected “pediatrics” regardless of intention to subspecialize) vs all other specialties (those who selected any other specialty choice). We created a 9-category variable to assess how loan repayment program (LRP) participation plans moderate pediatrics specialty choice by education debt reported at graduation: no education debt (therefore, no LRP participation plan) and 4 groupings of education debt ($1000-$149 999, $150 000-$249 999, ≥$250 000, and missing amount but indicated education debt), each further subdivided into plan or no plan for LRP participation. We examined 2 measures about respondents’ pediatrics clerkship experience: quality (response option range, 1 [poor] to 4 [excellent]) and extent of agreement with “faculty provided effective teaching during the clerkship,” dichotomized as agree (strongly agree and agree responses) and disagree or neutral (strongly disagree, disagree, and neutral responses). We created indicators for respondents who selected (vs did not select) each of patient care and research as planned career activities.

Using AAMC Organizational Characteristics Database data,^28^ we created 3 indicators for characteristics of medical school attended: ownership (private or public); community based (yes [a school without an integrated teaching hospital, received full accreditation in 1972 or later, and is nonfederal]^28^ or no [all other schools]); and research-intensive (yes [schools ranked among the top 40 in 2016 according to federal research expenditures from direct federal grants and contracts for organized research]^28^ or no [all other schools]).

### Statistical Analysis

We used χ^2^ tests, analysis of variance, and correlation coefficients for bivariate associations and multivariable, multilevel logistic regression (nested by school) for independent associations with pediatrics (vs all other) specialty choice at graduation; we also examined this outcome in models stratified by gender. All analyses considered 2-sided *P* < .05 to be significant and were conducted using Stata, version 19 (StataCorp LLC).

## Results

Across all seven 2013-2014 through 2019-2020 matriculant cohorts, there were 134 759 study-eligible graduates; 104 025 (77% of 134 759) responded to the GQ specialty choice item. Our study sample of 101 579 graduates included 98% of all 104 025 GQ respondents (75% of all 134 759 study-eligible graduates) and excluded 24 GQ respondents with an undefined gender and 2422 with missing data for 1 or more other GQ variables examined (pediatrics clerkship quality, agreement with pediatrics faculty teaching effectiveness, each of patient care and research career activity plans, and education debt). Table 1 shows the characteristics of all 134 759 study-eligible graduates by study sample inclusion status. Women and White students were overrepresented among those included vs excluded from our study sample (52% [52 810 of 101 579] vs 44% [14 449 of 33 148] and 54% [55 027 of 101 579] vs 43% [14 404 of 33 180], respectively).

**Table 1.** Study Sample Derivation Among Study-Eligible US MD-Granting Medical School Graduates (N = 134 759)

Demographic characteristic	No. (%)^a^			*P* value
	Study-eligible population^b^	Graduates included in study sample	Graduates excluded from study sample	
Gender				
Men	67 468 (50)	48 769 (48)	18 699 (56)	<.001
Women	67 259 (50)	52 810 (52)	14 449 (44)	
Total^c^	134 727 (100)	101 579 (100)	33 148 (100)	
Total population	134 759 (100)	101 579 (100)	33 180 (100)	
Race and ethnicity^d^				
Asian (alone)	28 187 (21)	20 351 (20)	7836 (24)	<.001
URiM (alone or in combination)	24 723 (18)	17 256 (17)	7467 (23)	
White (alone)	69 431 (52)	55 027 (54)	14 404 (43)	
All other or unknown	12 418 (9)	8945 (9)	3473 (10)	
Academic year of matriculation				
2013-2014	19 337 (14)	14 347 (14)	4990 (15)	<.001
2014-2015	19 532 (14)	15 087 (15)	4445 (13)	
2015-2016	19 670 (15)	15 232 (15)	4438 (13)	
2016-2017	19 726 (15)	14 734 (15)	4992 (15)	
2017-2018	19 889 (15)	14 587 (14)	5302 (16)	
2018-2019	19 776 (15)	14 781 (15)	4995 (15)	
2019-2020^e^	16 829 (12)	12 811 (13)	4018 (12)	

Abbreviation: URiM, underrepresented in medicine.

^a^ Percentages shown are those of the column total for each variable and may not add up to 100 due to rounding.

^b^ All 2013-2014 through 2019-2020 US MD-granting school matriculants who graduated and were invited to complete the Association of American Medical Colleges Graduation Questionnaire through 2022-2023.

^c^ Thirty-two total graduates who reported an undefined gender were excluded from the total for gender.

^d^ URiM includes each of the following alone or in combination with another race or ethnicity: American Indian or Alaska Native; Black or African American; Hispanic, Latino, or of Spanish origin; and Native Hawaiian or Other Pacific Islander. All other includes non-URiM combinations of Asian, White, and other (selection of “other” response), and missing race or ethnicity.

^e^ The 2019-2020 matriculant cohort was relatively underrepresented compared with earlier matriculant cohorts among study-eligible graduates due to the shorter duration of follow-up (4 years) for this entire matriculant cohort compared with the earlier matriculant cohorts.

Table 2 shows the characteristics of all 101 579 study graduates grouped by specialty choice at graduation; 10% (10 031 of 101 579) chose pediatrics at graduation. As shown, most women chose specialties other than pediatrics at graduation (86% [45 350 of 52 810]). Among all study graduates, the proportion of those who chose pediatrics at graduation varied by each variable examined (each *P* < .001). Not shown, the proportion of graduates who chose pediatrics varied by medical school (N = 153; range, 3%-19%; median, 10%; *P* < .001). eTable 3 in Supplement 1 shows specialty choice by academic year of matriculation for graduates in our study sample with MSQ and GQ specialty choice data. Among these 69 952 graduates, there was a significant negative correlation between proportion of graduates who intended pediatrics at matriculation and year of matriculation (2013-2014: 12% [1229 of 10 527], 2019-2020: 10% [892 of 9221]; *r* = −0.97; *P* < .001). Across years, of all 7628 graduates who reported a pediatrics intention at matriculation, 3280 (43%) sustained this pediatrics intention at graduation.

**Table 2.** Characteristics of the Final Study Sample Grouped by Specialty Choice at Graduation (N = 101 579)

Variable	No. (%)^a^			*P* value
	Total	Pediatrics specialty choice at graduation	All other specialty choices at graduation	
Total sample	101 579 (100)	10 031 (100)	91 548 (100)	NA
Demographic characteristics				
Gender				
Men	48 769 (48)	2571 (26)	46 198 (50)	<.001
Women	52 810 (52)	7460 (74)	45 350 (50)	
Race and ethnicity^b^				
Asian (alone)	20 351 (20)	1763 (18)	18 588 (20)	<.001
URiM (alone or in combination)	17 256 (17)	1669 (17)	15 587 (17)	
White (alone)	55 027 (54)	5889 (59)	49 138 (54)	
All other/unknown	8945 (9)	710 (7)	8235 (9)	
Academic year of matriculation				
2013-2014	14 347 (14)	1625 (16)	12 722 (14)	<.001
2014-2015	15 087 (15)	1561 (16)	13 526 (15)	
2015-2016	15 232 (15)	1497 (15)	13 735 (15)	
2016-2017	14 734 (15)	1424 (14)	13 310 (15)	
2017-2018	14 587 (14)	1388 (14)	13 199 (14)	
2018-2019	14 781 (15)	1328 (13)	13 453 (15)	
2019-2020	12 811 (13)	1208 (12)	11 603 (13)	
Career plan at matriculation				
Specialty intention reported at matriculation				
Pediatrics	7628 (8)	3280 (33)	4348 (5)	<.001
All other specialties	62 324 (61)	3938 (39)	58 386 (64)	
Missing specialty choice	31 627 (31)	2813 (28)	28 814 (31)	
Educational experiences and setting				
Quality of pediatrics clerkship, per point increase in rating, mean (SD)^c^	3.40 (0.78)	3.74 (0.55)	3.36 (0.79)	<.001
Faculty provided effective teaching during the pediatrics clerkship^d^				
Disagree or neutral	10 610 (10)	305 (3)	10 305 (11)	<.001
Agree	90 969 (90)	9726 (97)	81 243 (89)	
School ownership				
Private	38 715 (38)	3656 (36)	35 059 (38)	<.001
Public	62 864 (62)	6375 (64)	56 489 (62)	
Community-based medical school^e^				
No	87 037 (86)	8478 (85)	78 559 (86)	<.001
Yes	14 542 (14)	1553 (15)	12 989 (14)	
Research-intensive medical school^f^				
No	70 369 (69)	7143 (71)	63 226 (69)	<.001
Yes	31 210 (31)	2888 (29)	28 322 (31)	
Career plans at graduation				
Planned career activities include patient care				
No	2557 (3)	97 (1)	2460 (3)	<.001
Yes	99 022 (97)	9934 (99)	89 088 (97)	
Planned career activities include research				
No	51 001 (50)	5768 (58)	45 233 (49)	<.001
Yes	50 578 (50)	4263 (43)	46 315 (51)	
Plan for LRP participation by education debt reported at graduation^g^				
No plan for LRP participation				<.001
No debt	25 967 (26)	2475 (25)	23 492 (26)	
$1000-$149 999	15 089 (15)	1400 (14)	13 689 (15)	
$150 000-$249 999	13 964 (14)	1233 (12)	12 731 (14)	
≥$250 000	9091 (9)	580 (6)	8511 (9)	
Missing debt amount	1397 (1)	118 (1)	1279 (1)	
Plan for LRP participation				
$1000-$149 999	5586 (6)	697 (7)	4889 (5)	
$150 000-$249 999	14 053 (14)	1825 (18)	12 228 (13)	
≥$250 000	15 438 (15)	1590 (16)	13 848 (15)	
Missing debt amount	994 (1)	113 (1)	881 (1)	

Abbreviations: LRP, loan repayment program; NA, not applicable; URiM, underrepresented in medicine.

^a^ Percentages shown are those of the column total for each variable and may not add up to 100 due to rounding.

^b^ URiM includes each of the following alone or in combination with another race or ethnicity: American Indian or Alaska Native; Black or African American; Hispanic, Latino, or of Spanish origin; and Native Hawaiian or Other Pacific Islander. All other includes non-URiM combinations of Asian, White, and other (selection of “other” response), and missing race or ethnicity.

^c^ Four-point rating for quality of pediatrics clerkship: 1 = poor, 2 = fair, 3 = good, 4 = excellent.

^d^ Agree includes respondents who selected agree or strongly agree. Disagree or neutral includes respondents who selected disagree, strongly disagree, or neutral.

^e^ Community-based medical schools (yes group) were defined as those that do not have an integrated teaching hospital, received full accreditation in 1972 or later, and are nonfederal.

^f^ Research-intensive medical schools (yes group) were defined as those ranked among the top 40 in 2016 according to federal research expenditures from direct federal grants and contracts for organized research.

^g^ Bivariate analysis results for pediatrics specialty choice at graduation and each of plan for LRP participation and education debt are shown in eTables 1 and 2 in Supplement 2.

Table 3 shows the multilevel logistic regression model results among all 101 579 graduates of 153 medical schools. Among demographic variables, odds of pediatrics specialty choice at graduation were higher for women (vs men; adjusted odds ratio [AOR], 2.34 [95% CI, 2.23-2.46]); odds were lower for those of Asian (AOR, 0.86 [95% CI, 0.81-0.91]), URiM (AOR, 0.84 [95% CI, 0.79-0.89]), and all other or unknown race and ethnicity (AOR, 0.78 [95% CI, 0.71-0.85]) (each vs White); and for more recent matriculants (each vs 2013-2014; 2014-2015: AOR, 0.87 [95% CI, 0.80-0.94]; 2015-2016: AOR, 0.83 [95% CI, 0.77-0.90]; 2016-2017: AOR, 0.78 [95% CI, 0.72-0.84]; 2017-2018: AOR, 0.75 [95% CI, 0.69-0.82]; 2018-2019: AOR, 0.72 [95% CI, 0.66-0.78]; 2019-2020: AOR, 0.72 [95% CI, 0.66-0.79]).

**Table 3.** Multilevel Logistic Regression Model Results (N = 101 579)

Variable	AOR (95% CI)	*P* value
Demographic characteristics		
Gender		
Men	1.00 [Reference]	NA
Women	2.34 (2.23-2.46)	<.001
Race and ethnicity^a^		
Asian (alone)	0.86 (0.81-0.91)	<.001
URiM (alone or in combination)	0.84 (0.78-0.89)	<.001
White (alone)	1.00 [Reference]	NA
All other or unknown	0.78 (0.71-0.85)	<.001
Academic year of matriculation		
2013-2014	1.00 [Reference]	NA
2014-2015	0.87 (0.80-0.94)	.001
2015-2016	0.83 (0.77-0.90)	<.001
2016-2017	0.78 (0.72-0.84)	<.001
2017-2018	0.75 (0.69-0.82)	<.001
2018-2019	0.72 (0.66-0.78)	<.001
2019-2020	0.72 (0.66-0.79)	<.001
Career plan at matriculation		
Specialty intention reported at matriculation		
All other specialties	1.00 [Reference]	NA
Pediatrics	9.21 (8.68-9.76)	<.001
Missing specialty choice	1.51 (1.37-1.77)	<.001
Educational experiences and setting		
Quality of pediatrics clerkship, per unit increase^b^	2.22 (2.13-2.32)	<.001
Faculty provided effective teaching during the pediatrics clerkship^c^		
Disagree or neutral	1.00 [Reference]	NA
Agree	1.56 (1.37-1.77)	<.001
School ownership		
Private	1.00 [Reference]	NA
Public	1.03 (0.96-1.11)	.43
Community-based medical school^d^		
No	1.00 [Reference]	NA
Yes	1.07 (0.98-1.18)	.15
Research-intensive medical school^e^		
No	1.00 [Reference]	NA
Yes	0.82 (0.76-0.89)	<.001
Career plans at graduation		
Planned career activities include patient care		
No	1.00 [Reference]	NA
Yes	2.19 (1.77-2.71)	<.001
Planned career activities include research		
No	1.00 [Reference]	NA
Yes	0.75 (0.72-0.79)	<.001
Plan for LRP participation by education debt reported at graduation		
No plan for LRP participation		
No debt	1.00 [Reference]	NA
$1000-$149 999	0.94 (0.87-1.01)	.10
$150 000-$249 999	0.89 (0.83-0.97)	.005
≥$250 000	0.65 (0.59-0.72)	<.001
Missing debt amount	0.90 (0.73-1.10)	.31
Plan for LRP participation		
$1000-$149 999	1.23 (1.11-1.35)	<.001
$150 000-$249 999	1.28 (1.19-1.37)	<.001
≥$250 000	1.06 (0.99-1.15)	.10
Missing debt amount	1.19 (0.96-1.48)	.12

Abbreviations: AOR, adjusted odds ratio; LRP, loan repayment program; NA, not applicable; URiM, underrepresented in medicine.

^a^ URiM includes each of the following alone or in combination with another race or ethnicity: American Indian or Alaska Native; Black or African American; Hispanic, Latino, or of Spanish origin; and Native Hawaiian or Other Pacific Islander. All other includes non-URiM combinations of Asian, White, and other (selection of “other” response), and missing race or ethnicity.

^b^ Four-point rating for quality of pediatrics clerkship: 1 = poor, 2 = fair, 3 = good, 4 = excellent.

^c^ Agree includes respondents who selected agree or strongly agree. Disagree or neutral includes respondents who selected disagree, strongly disagree, or neutral.

^d^ Community-based medical schools (yes group) were defined as those that do not have an integrated teaching hospital, received full accreditation in 1972 or later, and are nonfederal.

^e^ Research-intensive medical schools (yes group) were defined as those ranked among the top 40 in 2016 according to federal research expenditures from direct federal grants and contracts for organized research.

Regarding career plan at matriculation, odds of pediatrics specialty choice at graduation were higher for those who reported (vs did not report) pediatrics specialty intention at matriculation (AOR, 9.21 [95% CI, 8.68-9.76]) and those missing specialty intention at matriculation (AOR, 1.51 [95% CI, 1.37-1.77]). Regarding educational experiences and setting, odds of pediatrics specialty choice at graduation were higher for those who rated the quality of their pediatrics clerkship more highly (AOR, 2.22 [95% CI, 2.13-2.32]; per unit increase in quality) and agreed (vs disagreed or neutral) that faculty provided effective teaching during their pediatrics clerkship (AOR, 1.56 [95% CI, 1.37-1.77]); odds were lower for those who attended research-intensive (vs non–research-intensive) schools (AOR, 0.82 [95% CI, 0.76-0.89]). We observed no difference in odds for those who attended publicly vs privately owned schools or community-based vs non–community-based schools (*P* > .05).

Regarding career plans at graduation, odds of pediatrics specialty choice at graduation were higher for those whose career plans included (vs did not include) patient care (AOR, 2.19 [95% CI, 1.77-2.71]), and for those with education debt of $1000 to $149 999 or $150 000 to $259 999 who planned for LRP participation (AOR, 1.23 [95% CI, 1.11-1.35] and 1.28 [95% CI, 1.19-1.37], respectively) (each vs those without education debt); odds were lower for those whose career plans included (vs did not include) research (AOR, 0.75 [95% CI, 0.72-0.79]) and for those with education debt of $150 000 to $249 999 or $250 000 or more who did not plan for LRP participation (AOR, 0.89 [95% CI, 0.83-0.97] and 0.65 [95% CI, 0.59-0.72], respectively) (each vs those without education debt).

Table 4 shows the results of gender-stratified multilevel logistic regression models. As shown, the direction and magnitude of the AORs were similar among men and among women for all variables examined. Differences in statistical significance by gender were observed for 2014-2015 (vs 2013-2014) matriculants (men: AOR, 0.89 [95% CI, 0.77-1.02]; women: AOR, 0.86 [95% CI, 0.78-0.94]), those with $150 000 to $249 999 of education debt who did not plan for LRP participation (men: AOR, 0.85 [95% CI, 0.73-0.98]; women: AOR, 0.92 [95% CI, 0.84-1.01]), and those with $250 000 or more of education debt who planned for LRP participation (men: AOR, 1.23 [95% CI, 1.07-1.40]; women: AOR, 1.01 [95% CI, 0.92-1.10]) (each vs those without education debt).

**Table 4.** Multilevel Logistic Regression Model Results Stratified by Gender (48 769 Men and 52 810 Women)

Variable	Men		Women	
	AOR (95% CI)	*P* value	AOR (95% CI)	*P* value
Demographic characteristics				
Race and ethnicity^a^				
Asian (alone)	0.77 (0.69-0.87)	<.001	0.89 (0.83-0.95)	.001
URiM (alone or in combination)	0.83 (0.73-0.93)	.004	0.84 (0.78-0.91)	<.001
White (alone)	1.00 [Reference]	NA	1.00 [Reference]	NA
All other or unknown	0.77 (0.66-0.91)	.002	0.78 (0.70-0.87)	<.001
Academic year of matriculation				
2013-2014	1.00 [Reference]	NA	1.00 [Reference]	NA
2014-2015	0.89 (0.77-1.02)	.10	0.86 (0.78-0.94)	.002
2015-2016	0.82 (0.71-0.96)	.01	0.83 (0.76-0.92)	<.001
2016-2017	0.86 (0.74-1.00)	.05	0.74 (0.67-0.83)	<.001
2017-2018	0.76 (0.65-0.88)	<.001	0.75 (0.68-0.83)	<.001
2018-2019	0.70 (0.60-0.82)	<.001	0.72 (0.66-0.80)	<.001
2019-2020	0.71 (0.61-0.84)	<.001	0.72 (0.65-0.80)	<.001
Career plan at matriculation				
Specialty intention at matriculation				
All other specialties	1.00 [Reference]	NA	1.00 [Reference]	NA
Pediatrics	12.32 (10.94-13.87)	<.001	8.48 (7.93-9.07)	<.001
Missing specialty choice	1.34 (1.22-1.48)	<.001	1.59 (1.49-1.70)	<.001
Educational experiences and setting				
Quality of pediatrics clerkship, per unit increase^b^	2.61 (2.39-2.85)	<.001	2.09 (1.98-2.20)	<.001
Faculty provided effective teaching during the pediatrics clerkship				
Disagree or neutral	1.00 [Reference]	NA	1.00 [Reference]	NA
Agree	1.55 (1.20-2.01)	.001	1.56 (1.34-1.81)	<.001
School ownership				
Private	1.00 [Reference]	NA	1.00 [Reference]	NA
Public	1.00 (0.89-1.11)	.96	1.04 (0.96-1.12)	.34
Community-based medical school^c^				
No	1.00 [Reference]	NA	1.00 [Reference]	NA
Yes	1.01 (0.87-1.17)	.94	1.10 (1.00-1.22)	.06
Research-intensive medical school^d^				
No	1.00 [Reference]	NA	1.00 [Reference]	NA
Yes	0.89 (0.79-1.00)	.06	0.80 (0.73-0.87)	<.001
Career plans at graduation				
Planned career activities include patient care				
No	1.00 [Reference]	NA	1.00 [Reference]	NA
Yes	1.74 (1.25-2.43)	.001	2.49 (1.89-3.27)	<.001
Planned career activities include research				
No	1.00 [Reference]	NA	1.00 [Reference]	NA
Yes	0.83 (0.76-0.90)	<.001	0.72 (0.68-0.76)	<.001
Plan for LRP participation by education debt reported at graduation				
No plan for LRP participation				
No debt	1.00 [Reference]	NA	1.00 [Reference]	NA
$1000-$149 999	0.91 (0.79-1.05)	.20	0.95 (0.87-1.04)	.28
$150 000-$249 999	0.85 (0.73-0.98)	.03	0.92 (0.84-1.01)	.07
≥$250 000	0.63 (0.53-0.76)	<.001	0.67 (0.59-0.76)	<.001
Missing debt amount	0.76 (0.49-1.19)	.23	0.94 (0.74-1.19)	.61
Plan for LRP participation				
$1000-$149 999	1.35 (1.11-1.63)	.002	1.17 (1.04-1.31)	.008
$150 000-$249 999	1.38 (1.21-1.58)	<.001	1.23 (1.13-1.34)	<.001
≥$250 000	1.23 (1.07-1.40)	.003	1.01 (0.92-1.10)	.91
Missing debt amount	1.38 (0.86-2.21)	.18	1.14 (0.89-1.46)	.30

Abbreviations: AOR, adjusted odds ratio; LRP, loan repayment program; NA, not applicable; URiM, underrepresented in medicine.

^a^ URiM includes each of the following alone or in combination with another race or ethnicity: American Indian or Alaska Native; Black or African American; Hispanic, Latino, or of Spanish origin; and Native Hawaiian or Other Pacific Islander. All other includes non-URiM combinations of Asian, White, and other (selection of “other” response), and missing race or ethnicity.

^b^ Four-point rating for quality of pediatrics clerkship: 1 = poor, 2 = fair, 3 = good, 4 = excellent.

^c^ Community-based medical schools (yes group) were defined as those that do not have an integrated teaching hospital, received full accreditation in 1972 or later, and are nonfederal.

^d^ Research-intensive medical schools (yes group) were defined as those ranked among the top 40 in 2016 according to federal research expenditures from direct federal grants and contracts for organized research.

## Discussion

We examined 7 cohorts of US MD matriculants who progressed through medical school and graduated (72% of all 142 059 US MD graduates from 2016-2017 through 2022-2023^19^) during a period of overall increase in the numbers of individuals matched to pediatrics positions for GME but also decreasing numbers of US MD graduates choosing pediatrics. Several key study findings may inform efforts to strengthen the emerging pediatrics workforce before and during medical school for US MD matriculants.

To our knowledge, this is the first study to examine gender-stratified models of pediatrics specialty choice at graduation. Among contemporary US MD student cohorts, Table 4 highlights many similarities in pediatrics specialty choice determinants among men and among women. Although women remained more likely than men to choose pediatrics at graduation, most women in our study chose specialties other than pediatrics at graduation (86% of 52 810). We speculate that various specialty-specific efforts over the past decade to increase the gender diversity of their workforces may be contributory, at least in part,^29^ as women entering and progressing through medical school may now view more specialties as feasible career options. In addition, our findings regarding decreased odds of URiM GQ respondents choosing pediatrics are similar to findings for 2005 to 2010 national GQ cohorts.^23^ The long-standing nature of this finding is concerning in the context of ongoing efforts to address current and anticipated future pediatrics workforce needs nationally.^13,30,31,32^ Further research to identify other potentially contributory variables, beyond those included in our study, is warranted.

Notably, pediatrics specialty intention at matriculation was the most strongly associated with pediatrics specialty choice at graduation in our entire sample and in gender-stratified models. According to recent aggregate data for a single year of GQ respondents, across all specialties, the durability at graduation of a particular specialty choice at matriculation is about 28%.^33^ This proportion ranged widely across specialties, with the percentage for pediatrics (43%) being among the highest of all specialties examined^33^ (aligning with our multiyear observation of 43%). Efforts to better understand contributors to the development of specialty preferences before medical school entry may be important for the pediatrics workforce.^34^ Our finding on specialty choice at matriculation strongly supports a recommendation to increase exposure to the discipline of pediatrics at “various critical touchpoints including high school and college.”^35^

Medical school exposures and experiences were independently associated with pediatrics specialty choice at graduation, suggesting opportunities for interventions in UME. For example, our observations add further evidence regarding the role of core clerkship experiences in specialty choice decisions.^14,24,25^ Unlike previous studies, however, our observations derive from models that controlled for specialty intention at matriculation. In this regard, our observations about the role of extrinsic factors during medical school align well with results of a recent qualitative study on the process of career decision-making among students with early career interest in pediatrics.^34^ It is also worth noting that the current structure of medical school curricula may favor study of adults in the preclinical and clinical stages over exposure to children because adults are the default model for curriculum building. This can make it challenging for UME pediatric advisors and clerkship directors to expose students throughout the curriculum (beyond the core pediatrics clerkship per se) to the breadth of pediatric medicine^36,37^; identifying potential opportunities to do so may be worthwhile for pediatrics educators to consider locally.

Ours is also the first study, to our knowledge, to examine education debt in the context of plans for LRP participation among US MD students planning pediatrics careers. Our findings suggest that planning to participate in LRPs may influence the association between graduates’ education debt and pediatrics specialty choice, particularly among those with relatively higher levels of debt, extending the work of others who examined this association among Doctor of Osteopathic Medicine graduates and primary care specialty choice.^38^ Working closely with students who may assume high levels of education debt so they are fully informed about LRPs and other debt management options might be particularly important early in their UME trajectories to ensure that students do not prematurely decide against particular specialties, such as pediatrics, based on an incomplete understanding of their debt management and/or reduction options. With the caveat that recent legislation may have effects on student borrowing, including those in medical education programs,^39^ recent data indicate that, among practicing US medical graduate physicians, the median time to repay education debt was 8.3 years or less for all specialty groups examined, including 7.9 years for primary care specialties.^39^ Additional data collection will be needed to discern how this legislation might impact pediatrics specialty choice specifically among students graduating with high levels of education debt.

Finally, our observation that the odds of pediatrics specialty choice at graduation were lower for GQ respondents whose career plans included research suggests that students interested in research may not see pediatrics as a good “fit”; the physician-scientist workforce pipeline in pediatrics has been a concern nationally.^40,41^ Earlier efforts to raise student awareness of research opportunities in pediatrics during UME and GME seem warranted. Faculty organizers of medical school Pediatrics Interest Groups^42^ may want to ensure their activities include a focus on pediatrics research opportunities^10^ and guidance for students seeking these opportunities during medical school,^43^ and information about continued research opportunities during pediatrics GME. The American Board of Pediatrics offers 2 research pathways to eligibility for certification,^41^ and according to recent AAMC GME Track data,^44^ of 210 responding pediatrics programs, 32 had required dedicated research rotations and 175 had optional dedicated research rotations for program trainees (personal written communication, Douglas Grbic, PhD, August 15, 2025).

### Limitations

This study has some limitations. Specialty choices offered in recent MSQs and GQs were expanded to include “internal medicine/pediatrics” and “child neurology.” As these 2 choices were not available options for all GQ respondents in our study sample, those who completed the MSQ and GQ in the most recent cohorts when these choices were available were included in the “all other specialties” group of the study outcome. Thus, our identification of students whose specialty choice at graduation was pediatrics may underestimate the total number of students in our study planning for pediatrics-related specialties. However, based on NRMP data,^5,6^ the decrease in pediatrics specialty choice at graduation among US seniors that we observed would still be evident if an expanded definition of “pediatrics” specialty choice including all pediatrics-related specialty choices were used; 2015 and 2024 NRMP data, provided in eTable 4 in Supplement 1, suggest that the decreasing number of US seniors matching to pediatrics programs over the decade was not due to concordant increases in the numbers of US seniors matching to other pediatrics-related programs, rather, the total number of US seniors who matched to all pediatrics-related programs decreased over this decade by 15%.

We also note that other variables not included in our study may be contributory to our outcome (eg, role of Pediatrics Interest Groups^42^ in sustaining and increasing interest in pediatrics and school-specific aspects of pediatrics clerkships^34,36,45^). Our results may not generalize to Doctor of Osteopathic Medicine or international medical graduates (19% and 22%, respectively, of the US Accreditation Council for Graduate Medical Education–accredited pediatrics residency workforce).^46^

## Conclusions

In this cohort study of 7 US MD matriculant cohorts, we identified variables independently associated with pediatrics specialty choice at medical school graduation. Although the proportions of matriculants who had chosen pediatrics at graduation decreased during our study period, a recent uptick in the number of US MD seniors matched to categorical pediatrics positions occurred in 2025 compared with 2024.^5^ Moreover, the number of medical school graduates of all types entering the pediatrics workforce has continued to increase, with greater representation of Doctor of Osteopathic Medicine and international medical graduates. With needs across the medical care continuum and a limited supply of trainees, our study highlights opportunities to sustain and promote interest in pediatrics among US MD matriculants.

## Notes

Supplement 1. **eTable 1.** Bivariate association with pediatrics specialty choice at graduation and plan for loan repayment program participation, among indebted graduates in the study sample (N=75,612) **eTable 2.** Bivariate association with pediatrics specialty choice at graduation and education debt reported at graduation among the entire study sample (N=101,579) **eTable 3.** Specialty choice by academic year of matriculation, among all US MD matriculant-respondents in the study with both MSQ and GQ data for specialty choice (N=69,952) **eTable 4.** US MD seniors matched to PGY-1 positions in all pediatrics-related programs from 2015 to 2024

Supplement 2.
Data Sharing Statement
