# Supplementary material for: Pediatrics Specialty Choice Among Matriculants of MD-Granting US Medical Schools
Source: JAMA Netw Open. 2026 Mar 4;9(3):e260603. doi: 10.1001/jamanetworkopen.2026.0603 (PMC12961512; doi:10.1001/jamanetworkopen.2026.0603)
Supplement: Supplement 1. — eTable 1. Bivariate association with pediatrics specialty choice at graduation and plan for loan repayment program participation, among indebted graduates in the study sample (N=75,612) eTable 2. Bivariate association with pediatrics specialty choice at graduation and education debt reported at graduation among the entire study sample (N=101,579) eTable 3. Specialty choice by academic year of matriculation, among all US MD matriculant-respondents in the study with both MSQ and GQ data for specialty choice (N=69,952) eTable 4. US MD seniors matched to PGY-1 positions in all pediatrics-related programs from 2015 to 2024 [file jamanetwopen-e260603-s001.pdf]

Supplemental Online Content

Pineda A, Vinci RJ, Turner A, et al. Pediatrics specialty choice among matriculants of MD-granting US medical schools. *JAMA Netw Open*. 2026;9(3):e260603. doi:10.1001/jamanetworkopen.2026.0603

eTable 1. Bivariate association with pediatrics specialty choice at graduation and plan for loan repayment program participation, among indebted graduates in the study sample (N=75,612)

eTable 2. Bivariate association with pediatrics specialty choice at graduation and education debt reported at graduation among the entire study sample (N=101,579)

eTable 3. Specialty choice by academic year of matriculation, among all US MD matriculant-respondents in the study with both MSQ and GQ data for specialty choice (N=69,952)

eTable 4. US MD seniors matched to PGY-1 positions in all pediatrics-related programs from 2015 to 2024

This supplemental material has been provided by the authors to give readers additional information about their work.

© 2026 Pineda A et al. *JAMA Network Open*.

eTable 1. Bivariate association with pediatrics specialty choice at graduation and plan for loan repayment program participation, among indebted graduates in the study sample (N=75,612).

Plan for loan repayment program participation	Total No. (%)	Pediatrics specialty choice at graduation No. (%)	All other specialty choices at graduation No. (%)	p <.001
No	39,541 (100)	3,331 (8)	36,210 (92)	
Yes	36,071 (100)	4,225 (12)	31,846 (88)	

© 2026 Pineda A et al. *JAMA Network Open*.

eTable 2. Bivariate association with pediatrics specialty choice at graduation and education debt reported at graduation among the entire study sample (N=101,579).

Education debt reported at graduation	Total No. (%)	Pediatrics specialty choice at graduation No. (%)	All other specialty choices at graduation No. (%)	
No debt	25,967 (100)	2,475 (10)	23,492 (90)	p <.001
$1,000-$149,000	20,675 (100)	2,097 (10)	18,578 (90)	
$150,000-$249,000	28,017 (100)	3,058 (11)	24,959 (89)	
$250,000+	24,529 (100)	2,170 (9)	22,359 (91)	
Missing amount (among indebted graduates)	2,391 (100)	231 (10)	2,160 (90)	

© 2026 Pineda A et al. *JAMA Network Open*.

eTable 3. Specialty choice by academic year of matriculation, among all US MD matriculant-respondents in the study with both MSQ and GQ data for specialty choice (N=69,952).

Academic year of matriculation	Total (column %)	A[a]: No. (row %) of respondents with pediatrics specialty choice in both the MSQ and GQ	B[a]: No. (row %) of respondents with pediatrics specialty choice in the MSQ and other specialty choice in the GQ	C: No. (row %) of respondents with other (non-pediatrics) specialty choice in the MSQ and pediatrics specialty choice in the GQ	D: No. (row %) of respondents with other (non-pediatrics) specialty choice in both the MSQ and GQ
2013-14	10,527 (15.0)	528 (5.0)	701 (6.7)	690 (6.6)	8,608 (81.8)
2014-15	10,974 (15.7)	565 (5.2)	723 (6.6)	610 (5.6)	9,076 (82.7)
2015-16	9,970 (14.3)	466 (4.7)	635 (6.4)	573 (5.8)	8,296 (83.2)
2016-17	9,204 (13.2)	405 (4.4)	607 (6.6)	514 (5.6)	7,678 (83.4)
2017-18	9,332 (13.3)	438 (4.7)	560 (6.0)	495 (5.3)	7,839 (84.0)
2018-19	10,724 (15.3)	467 (4.4)	641 (6.0)	537 (5.0)	9,079 (84.7)
2019-20	9,221 (13.2)	411 (4.5)	481 (5.2)	519 (5.6)	7,810 (84.7)
Total (row %)	69,952 (100)	3,280 (4.7)	4,348 (6.2)	3,938 (5.6)	58,386 (83.5)
Correlation between academic year of matriculation and percentage of respondents, p-value	-.52, p=.232	-.78, p=.037	-.89, p=.008	-.73, p=.062	0.98, p<.001

Abbreviations: MSQ, Matriculating Student Questionnaire; GQ, Graduation Questionnaire.

[a]The sum of column A and column B is the total number of MSQ respondents who reported a pediatrics intention at matriculation (total across all years=7,628 [3,280 + 4,348]). The proportion of MSQ respondents who reported an interest in pediatrics at matriculation (sum of column A and column B) decreased from 11.7% (1,229/10,527) in 2013-2014 to 9.7% (892/9,221) in 2019-2020; r = -.97, p <.001.

© 2026 Pineda A et al. *JAMA Network Open*.

eTable 4. US MD seniors matched to PGY-1 positions in all pediatrics-related programs from 2015 to 2024.

Program	2015[1] No. (% of all 16,932 USS matched to PGY-1 positions)	2024[2] No. (% of all 18,465 USS matched to PGY-1 positions)
Child Neurology	73 (0.4)	133 (0.7)
Medicine-Pediatrics	319 (1.9)	339 (1.8)
Pediatrics-Categorical	1889 (11.2)	1464 (7.9)
All other pediatrics-related programs: "Pediatrics-Anesthesiology", "Pediatrics-Emergency Medicine", "Pediatrics-Medical Genetics", "Pediatrics-Physical Medicine & Rehabilitation", "Pediatrics-Preliminary", "Pediatrics-Primary", and "Pediatrics/Psychiatry/Child Psychiatry"	94 (0.6)	87 (0.5)
**All pediatrics-related programs combined**	**2,375 (14.0)**	**2,023 (11.0)**

Abbreviations: PGY, post graduate year; USS, US MD Seniors.

[1]Table 10A. U.S. MD Seniors Matched to PGY-1 Positions by Specialty 2011-2015. National Resident Matching Program, Results and Data: 2015 Main Residency Match®. National Resident Matching Program, Washington, DC. 2015.

[2]Table 10A. U.S. MD Seniors Matched to PGY-1 Positions by Specialty 2020-2024. National Resident Matching Program, Results and Data: 2024 Main Residency Match®. National Resident Matching Program, Washington, DC. 2024.

eTable 4 shows 2015 and 2024 NRMP data for total numbers of US seniors matched to post-graduate year one positions in all pediatrics-related specialty programs. As shown, from 2015 to 2024, the total number of US seniors matched to positions in all specialties increased by 9% (2015: 16,932; 2024: 18,465). In 2015, 2,375 US seniors matched to positions in all types of pediatrics programs (14% of all 16,932 US seniors matched to positions in all specialties). In 2024, 2,023 US seniors matched to positions in all types of pediatrics programs (11% of all 18,465 US seniors matched to positions in all specialties). These data suggest that the declining number of US seniors matching to pediatrics programs over the decade was not due to concordant increases in the numbers of US seniors matching to other pediatrics-related programs; rather, the total number of US seniors who matched to all pediatrics-related programs declined over this decade by 15%.

© 2026 Pineda A et al. *JAMA Network Open*.
