# Supplementary material for: Pediatrics Specialty Choice Among Matriculants of MD-Granting US Medical Schools
Source: JAMA Netw Open. 2026 Mar 4;9(3):e260603. doi: 10.1001/jamanetworkopen.2026.0603 (PMC12961512; doi:10.1001/jamanetworkopen.2026.0603)
Supplement: Supplement 2. — Data Sharing Statement [file jamanetwopen-e260603-s002.pdf]

Data Sharing Statement

Pineda. Pediatrics Specialty Choice Among Matriculants of MD-Granting US Medical Schools. *JAMA Netw Open*. Published March 04, 2026. doi:10.1001/jamanetworkopen.2026.0603

Data
Data available: No

Additional Information
Explanation for why data not available: Study data are sensitive and proprietary, and therefore, are not available for public sharing by the authors. Access to the data was granted to the authors only for the purposes of the described study.
